# Systematic Study of the Immune Components after Ischemic Stroke Using CyTOF Techniques

**DOI:** 10.1155/2020/9132410

**Published:** 2020-08-28

**Authors:** Yaning Li, Yan Wang, Yang Yao, Brian B. Griffiths, Liangshu Feng, Tao Tao, Feng Wang, Baohui Xu, Creed M. Stary, Heng Zhao

**Affiliations:** ^1^Department of Neurosurgery, School of Medicine, Stanford University, Stanford, CA 94305, USA; ^2^Department of Anesthesiology, Perioperative and Pain Medicine, School of Medicine, Stanford University, CA 94305, USA; ^3^Center for Microbiota and Immunological Diseases, Shanghai General Hospital, Shanghai Institute of Immunology, Shanghai Jiao Tong University School of Medicine, Shanghai 200025, China; ^4^Division of Vascular Surgery, School of Medicine, Stanford University, Stanford, CA 94305, USA

## Abstract

Stroke induces a robust inflammatory response. However, it still lacks a systematic view of the various immune cell types due to the limited numbers of fluorophore used in the traditional FACS technique. In our current study, we utilized the novel technique mass cytometry (CyTOF) to analyze multiple immune cell types. We detected these immune cells from the ischemic brain, peripheral blood, spleen, and bone marrow at different time courses after stroke. Our data showed (1) dynamic changes in the immune cell numbers in the ischemic brain and peripheral organs. (2) The expression levels of cell surface markers indicate the inflammation response status after stroke. Interestingly, CD62L, a key adhesion molecule, regulates the migration of leukocytes from blood vessels into secondary lymphoid tissues and peripheral tissues. (3) A strong leukocyte network across the brain and peripheral immune organs was identified using the R program at day 1 after ischemia, suggesting that the peripheral immune cells dramatically migrated into the ischemic areas after stroke. This study provides a systematic, wide view of the immune components in the brain and peripheral organs for a deep understanding of the immune response after ischemic stroke.

## 1. Introduction

Stroke is one of the leading causes of human death worldwide [1]. Focal cerebral ischemia is the major type of stroke, leading to rapid neuronal injury. Stroke outcome is modulated by immune responses, which are characterized by neuroinflammation in the ischemic brain and immune suppression in the peripheral organs [2, 3]. Despite extensive studies in the past decades, a systematic picture of the immune response across the whole body is not fully understood.

Neuronal injury immediately after stroke triggers the resident microglia activity, followed by the infiltration of peripheral circulating leukocytes [4–8]. In contrast, lymphopenia occurs from a few hours to weeks in the peripheral immune organs [9–13]. We and others have reported that there are correlations between brain neuroinflammation and immunosuppression in the peripheral organs [14]. In our current study, we used the mass cytometry (CyTOF) technique to quantify the immune cell numbers in different organs from 1 day to 14 days after stroke and analyzed the cell surface marker expressions, which showed that CD62L might mediate some specific immune cell type, such as Tregs, recruitment during the subacute stroke status. In addition, the R program was used to predict the immune cell networks across organs at different timeframes. All of these findings aim to reveal a comprehensive picture of the immune response after stroke.

## 2. Materials and Methods

### 2.1. Animals and Surgery

Male C57BL/6J mice were used in this study (8–10 weeks, stock number 000664) and were purchased from Jackson Laboratories (Maine, USA). Five mice were housed in each cage at the Stanford animal facility under a 12 : 12 h light-dark cycle (lights on at 07:00 a.m.), at a temperature of 65–75°F and 40–60% humidity, with freely available food and water. All animal experiments were performed under the protocols based on the NIH Guidelines for Care and Use of Laboratory Animals. All experiments were performed in accordance with the ARRIVE (Animal Research Reporting In Vivo Experiments) Guidelines and approved by the Stanford Institutional Animal Care and Use Committee (IACUC). For the sham group and at each time point, samples from five mice were collected. Anesthesia was induced by 5% isoflurane and maintained by 1.5 to 2% isoflurane mixed with medical air with a facemask. Middle cerebral artery occlusion (MCAO) was induced as previously described [3, 8]. Briefly, after isolating the common carotid artery (CCA), external carotid arteries (ECA), and internal carotid arteries (ICA), the left MCA was occluded by gently inserting a silicone-coated 6-0 nylon suture (Doccol Corp., Sharon, MA) from the ECA into the MCA; occlusion was verified by laser Doppler. Reperfusion was achieved after 90 min of MCAO by suture withdrawal [13, 15]. Sham control group is a sham group that had been subjected to the stroke-induction procedure (without actually inducing stroke), aiming to rule out the effect of the procedure itself on the expression of or recovery from stroke. The mice in the sham group were euthanized on day 3.

### 2.2. Collection of Peripheral Blood, Bone Marrow, Spleen, and Ischemic Brains

The mice were euthanized at day 1, day 3, day 7, and day 14 after stroke. The ischemic brain tissue, peripheral blood, spleen, and bone marrow were collected, as described [16, 17] for CyTOF analysis. Mice with sham surgery underwent the same procedure without MCA occlusion. Before animal euthanization, peripheral blood (50 *μ*l) was collected from the tail vein of the animals into a tube containing 100 *μ*l of 10 mM EDTA at 1 day, 3 days, 7 days, and 14 days after stroke or from animals with sham surgery. After euthanization by an overdose of isoflurane, the right tibia bone and the spleen were removed and placed into cold RPMI 1640 (Life Technologies, Carlsbad, CA). After the mice were perfused with iced phosphate-buffered saline (PBS), the brains were collected and ischemic hemispheres were dissected.

### 2.3. Cell Preparations for CyTOF Immunostaining

All cell isolation procedures were performed either on ice or at 4°C. (A) The whole blood cells were used for immunostaining without further isolation. (B) To prepare the splenocytes, the spleen was minced, filtered through a 70 *μ*m cell strainer into 5 ml of RPMI 1640, and then centrifuged at 200 × g for 10 min. The cell pellet was resuspended, and red blood cells were lysed in 1 ml of ACK red blood cell lysis buffer; then, 2 ml of RPMI 1640 was added and centrifuged, and the pellet was resuspended in PBS for further staining. (C) Bone marrow cells in the tibia bone were flushed out by using a syringe containing 1 ml of RPMI 1640. The cells were centrifuged at 200 × g for 10 min and then resuspended in PBS for staining. (D) Each ischemic hemisphere was minced in RPMI 1640 and filtered through a 70 *μ*m cell strainer and adjusted to 7 ml, to which 3 ml of 90% Percoll (GE Healthcare, Chicago, IL) was added and mixed. 2 ml of 70% Percoll was loaded into the bottom of the cell suspension and centrifuged at 600 × g for 30 min. The interphase cells were collected, added to RPMI 1640, adjusted to a final volume of 5 ml, centrifuged at 200 × g for 10 min, and resuspended in PBS for further staining.

### 2.4. Live/Dead Staining

To discriminate the live cells from dead cells for mass cytometry, cisplatin was used to stain the dead cells, as it enters intracellularly via compromised cell membranes and labels all proteins. In brief, the whole blood and cells (1 − 2 × 10^6^ cells each sample) prepared from the brain, spleen, and bone marrow were washed with PBS and centrifuged at 300 × g for 5 min. The supernatant was discarded, and the cell pellet was stained in a 100 *μ*l solution of Cell-ID Cisplatin (Fluidigm Corp., S. San Francisco, CA) with a final concentration of 5 *μ*M cisplatin. After incubating at room temperature (RT) for 5 min, the staining was quenched with 500 *μ*l Maxpar Cell Staining Buffer (Fluidigm Corp.) and centrifuged to discard the supernatant.

### 2.5. Barcoding Procedure

In conventional flow cytometry, every sample is collected and stained separately, which raises the variability due to sample staining and machine analysis procedures. Thus, huge efforts for standardization and normalization are required to enhance the comparability between different samples. However, in mass cytometry, barcoding systems are used so that different samples can be combined as one multiplexed sample. Data obtained will then be subjected to debarcoding for individual sample analysis. The barcoding system reduces variability between samples and instrument sensitivity. The barcode system used in our study was previously established [18], with a commercially available kit. This 20-Plex Pd barcoding system provides 20 different barcodes, which allows us to label 20 samples and then pool them together for further processing as a single, multiplexed sample. The mass-tag barcoding agents label cell samples with a unique combination of three palladium (Pd) isotopes. To barcode samples, the samples need to be fixed and permeabilized. The cells were resuspended in 1 ml Fix I Buffer (Fluidigm Corp.) and incubated for 10 min at RT. The cells were centrifuged, and the cells in the pellet were permeabilized by washing with 1 ml Barcode Perm Buffer (Fluidigm Corp.). After fixation and permeabilization, each sample was resuspended in 800 *μ*l Barcode Perm Buffer (Fluidigm Corp.), added to barcodes, diluted in 100 *μ*l Barcode Perm Buffer, mixed, and incubated for 30 min at RT. The cells were centrifuged and washed twice with 1 ml of Maxpar Cell Staining Buffer. The resulting cell pellet was used for immunostaining.

### 2.6. Immunostaining

After barcoding, the cells were resuspended in 50 *μ*l Maxpar Cell Staining Buffer. Fc-Receptor (BioLegend, San Diego, CA) blocking solution was added then incubated for 10 min, and then 50 *μ*l of antibody cocktail was added to make a total of 100 *μ*l volume. The mixture was gently vortexed, incubated for 30 min at RT, and centrifuged, and the supernatant was discarded. The cell pellet was added to 500 *μ*l of intercalator solution (Fluidigm Corp.), gently vortexed, and incubated for 1 h at RT. The cells were washed by adding 1 ml of Maxpar Cell Staining Buffer and centrifuged. The cell pellet was resuspended with EQ Four Element Beads (Fluidigm Corp.) solution, and the cell concentration was adjusted to 2.5 − 5 × 10^5^/ml. The cells were injected into the CyTOF machine and processed for data acquirement (CyTOF2, Fluidigm Corp.). The list of antibodies is summarized in Table 1; all antibodies were purchased from Fluidigm Corp. The immune cell types identified by CyTOF in the brain and peripheral immune organs are listed in Table 2.

### 2.7. Data Analysis

The raw data were first calibrated using beads. The multiplexed samples were debarcoded by the Debarcoder software (Fluidigm Corp.) to separate different samples based on a single-cell algorithm, to provide individual data files. Then, the data were analyzed by using Cytobank [19] and SPADE [20]. The values were transformed to ArcSinh scales. Heat map data were generated using the ArcSinh ratio with the minimum value as control baseline using Cytobank https://www.cytobank.org [19, 21] to present the cell marker expression changes in different cell types. The expression level of each marker was normalized by dividing the maximal measured median intensity value.

### 2.8. R Program Analysis

Correlational matrices were generated using total cell numbers for each animal at various time points across the four organs, for each organ across the five time points, resulting in large correlational matrices. Any correlations that were -1 or 1 were thrown out. We used the Benjamini-Hochberg adjustment for multiple comparisons, then further restricted the cutoff value to only analyze correlations where *R*^2^ > 0.90. The data was loaded into R studio (v1.1.383; R v 3.4.3) and network graphs were generated using the iGraph package (v1.1.2) with a Fruchterman-Reingold clustering. Graphs were exported as .svg and edited in Inkscape for clarity. To present the immune cell-type network, the red lines indicate the positive correlations, while the black lines show the negative correlations. Among the various immune cell types, when they are connected by either a red line or black line, they were considered within the same network.

### 2.9. Statistical Analysis

Data are expressed as mean ± SD. Differences were considered statistically significant when *P* < 0.05. Parametric data from different groups were compared using 1-way ANOVA followed by Student–Newman–Keuls tests using GraphPad Prism. Two-way ANOVA was used when comparing groups with two variants (GraphPad Software, Inc., San Diego, CA, USA).

## 3. Results and Discussion

### 3.1. The Trend of Various Immune Cell Numbers at Different Time Points after Ischemic Stroke

**Figure 1:**
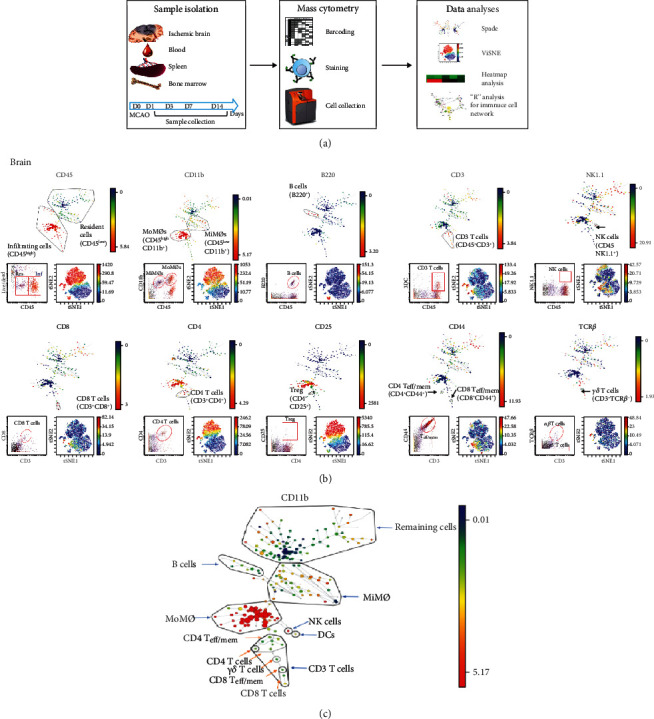
Characterization of the brain immune cell populations using mouse MCAO model by CyTOF. (a**)** Experimental workflow: mice with stroke and sham surgery were euthanized for tissue collections. The ischemic brain hemisphere, blood, spleen, and bone marrow were collected at days 1, 3, 7, and 14 after stroke, and immune cells were isolated and barcoded by a combination of three palladium (Pd) mass tags. Cells from the same tissue types were collected at the same time points, pooled, and stained using metal-labeled 17 antibodies against cell surface markers. The CyTOF data was analyzed using Cytobank and presented by SPADE, ViSNE, and standard dot pot. The marker intensity was analyzed using a heat map. The cell number correlations were analyzed using R programming language and presented as network. (b) Representative gating method. Cell surface markers used for cell-type identification including CD45, CD11b, B220, NK1.1, CD3, CD8, CD4, CD44, CD25, and TCR*β*. Illustrative SPADE tree (top), viSNE (bottom right), and standard dot pot (bottom left) to present identified immune cell types in the ischemic brain. In SPADE, the colored standards present the markers' median expression levels, and the node sizes show the cell numbers. In viSNE, the color gradients show the markers' intensity, and each dot represents a single cell. All of the figures are representative data on day 3 after stroke onset. (c) Illustrative SPADE identification of brain immune cells (day 3).

There are two major improvements for CyTOF compared to traditional FACS: first and foremost, the number of probes used in one reaction significantly increased. Secondly, the CyTOF procedure requires barcoding of each sample before staining (Figure 1(a)). All of the samples go through the same staining, washing, and permeabilization steps as a single multiplexed sample. This reduces the variations among samples, and the results are more consistent and accurate. In the current study, the samples from the sham group and various time points after stroke on set were collected and stained strictly following the CyTOF staining protocol. Then, the data were analyzed using both viSNE and SPADE (Figure 1(b)). viSNE presents a two-dimensional data; each individual cell shows a scatter plot to present the labeled marker expression level for each cell [22]. SPADE organizes cells into a hierarchy based on the related phenotype markers to identify cell types and shows the cellular heterogeneity [20].

As a result, we identified 11 leukocytes recruited in the ischemic brain (Figure 1(c)).

**Figure 2:**
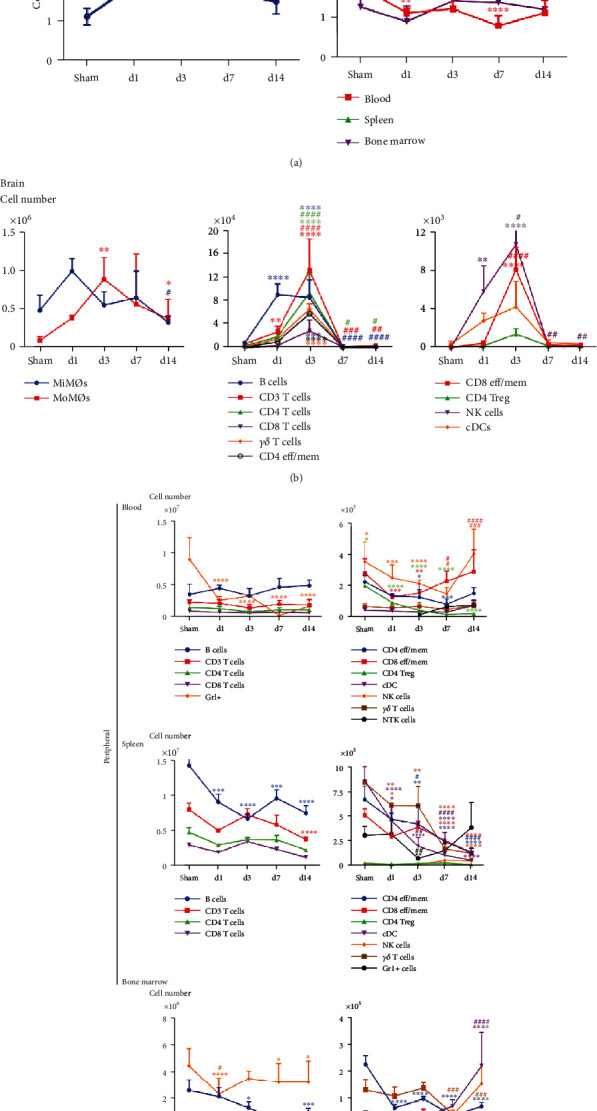
The trend of cell number changes in different tissues among acute, subacute, and chronic stages after MCAO in mice. (a) Total cell number changes in the brain, blood, spleen, and bone marrow from sham, day 1, day 3, day 7, and day 14. (b) Changes in various immune cell numbers in the sham group brain and day 1, day 3, day 7, and day 14 after stroke. Based on the cell numbers, the brain immune cells were divided into three different groups. The first group (Figure 2(b), left) includes MoMØs and MiMØs. *P* = 0.06 (MiMØs, sham control vs. day 1 group). The second group (Figure 2(b), middle) includes B cells, CD3 T cell, CD4 T cells, CD8 T cells, *γδ*T cells, and CD4 T_EFF/MEM_ cells. The third group (Figure 2(b), right) includes CD8 T_EFF/MEM_ cells, Treg cells, NK cells, and cDCs. (c) Changes in various immune cell numbers in the peripheral blood, spleen, and bone marrow from the sham group and day 1, day 3, day 7, and day 14 after stroke. Based on the cell numbers, cells were divided into two groups. The first group (on the left) includes B cells, CD3 T cell, CD4 T cells, CD8 T cells, and Gr1^+^ cells (in the spleen, GR1^+^ cells were listed in the second group). The second group (on the right) includes CD4 T_EFF/MEM_ cells, CD8 T_EFF/MEM_ cells, *γδ*T cells, Treg cells, NK cells, and cDCs. ^∗^Sham vs. different time points. ^#^1 d vs. various time points. ^∗^^/#^*P* < 0.05, ^∗∗^^/##^*P* < 0.01, ^∗∗∗^^/###^*P* < 0.001, and ^∗∗∗∗^^/####^*P* < 0.0001.

Consistent with previous data, CyTOF results showed that the total leukocyte numbers significantly increased in the brain after ischemic stroke but dropped in the peripheral blood, spleen, and bone marrow (Figure 2(a)). Across the time points, the total leukocyte population numbers in the ischemic brains were increased from day 1 and reached a peak at day 3 after ischemia (Figure 2(a)). We divided the brain immune cells into three groups, based on the cell numbers, for a clear review of each cell type (Figure 2(b)). First, MiMØs and MoMØs are the major immune components in the ischemic brain. There were approximately a half million resident microglia in the ischemic hemisphere, which quickly responded to ischemia and increased from day 1, while MoMØs increased from day 1 and peaked at day 3. In the subacute stage, the MoMØ numbers maintained at a relatively higher level (Figure 2(b), left). Notably, unlike most T cells (including CD4, CD8, and TEM cells), which reached peaks on day 3, B cells responded much faster to stroke damage and dramatically migrated into the brain from day 1 (Figure 2(b), middle). In addition, NK cells also entered the ischemic brain from day 1 and reached a peak on day 3 after stroke.

On the other hand, the total cells in the blood and spleen all experienced a significant decrease (Figures 2(a) and 2(c)). In the bone marrow, both Gr1^+^ and B cells showed significant decrease (Figure 2(c)).The detailed characterization of the immune cells using CyTOF are provided in supplementary data. (Supplementary Figure 1 for the blood, Supplementary Figure 2 for the spleen, and Supplementary Figure 3 for the bone marrow). The top channels show the SPADE analysis, and bottom/right channels show the viSNE analysis (supplementary data). In Figure 2, for better visualization, the cell types are depicted in two separated graphs based on cell numbers (10^5^ vs. 10^6^ or 10^7^). In our current study, we have seen a dramatic decrease of Gr1^+^ cells containing both monocytes and neutrophils from the acute phase in the peripheral blood, which further confirmed that the number of monocytes and neutrophils is closely related to stroke status. Previous studies indicated that leukocyte numbers could be used as markers to estimate the severity of ischemic stroke. As an inflammation marker, the monocyte counts could predict not only the first cerebral infarction [23] but also the recurrent ischemic events [24]. The total immune cells in the spleen had a significant decline on day 1 with the most dramatic decrease on day 3 after MCAO (Figure 2(b)). The leukocyte cell numbers in the peripheral blood were also taken as an indicator for stroke severity. For instance, data collected from a 3-month study in Chinese stroke patients showed that the ratio of lymphocytes to monocytes in the blood is a novel predictor for acute ischemic stroke [25]. The total white blood cell count and absolute neutrophil count were used as prognostic biomarkers in human intracerebral hemorrhage (ICH) [10]. The spleen has a critical connection with the ischemic brain during the stroke-induced inflammation process. It serves as a major reservoir for circulating immune cells and a crucial peripheral immune organ that mediates inflammation [26]. Rats splenectomized 2 weeks before permanent MCAO had more than an 80% decrease in infarction [27]. Although, with or without splenectomies, it did not significantly affect the cell number changes in the blood [27], the absolute spleen weight started to decrease from 24 h, and the most significant decline was seen around 50 h after MCAO [9]. In our current study, we have also seen a dramatic decrease of total immune cells in the spleen 1 day after MCAO, with the significant decrease appearing at day 3 (Figure 2(b)). This decrease is a systematic response, and all cell type numbers had declined. A previous report showed that various spleen immune cells, including NK cells, monocytes, and T cells, labeled by carboxyfluorescein diacetate succinimidyl ester (CFSE) can be detected in the ischemic brain [9], which shows that the spleen serves as a source of immune cells migrating into the brain and plays a role in neuroinflammation. All of these confirmed that lymphopenia happened in the peripheral organs and the decreased immune cell numbers may be used as predictors for stroke outcome.

### 3.2. The Expression Changes of Immune Cell Surface Markers Indicate the Possible Function and Status of Immune Cells

**Figure 3:**
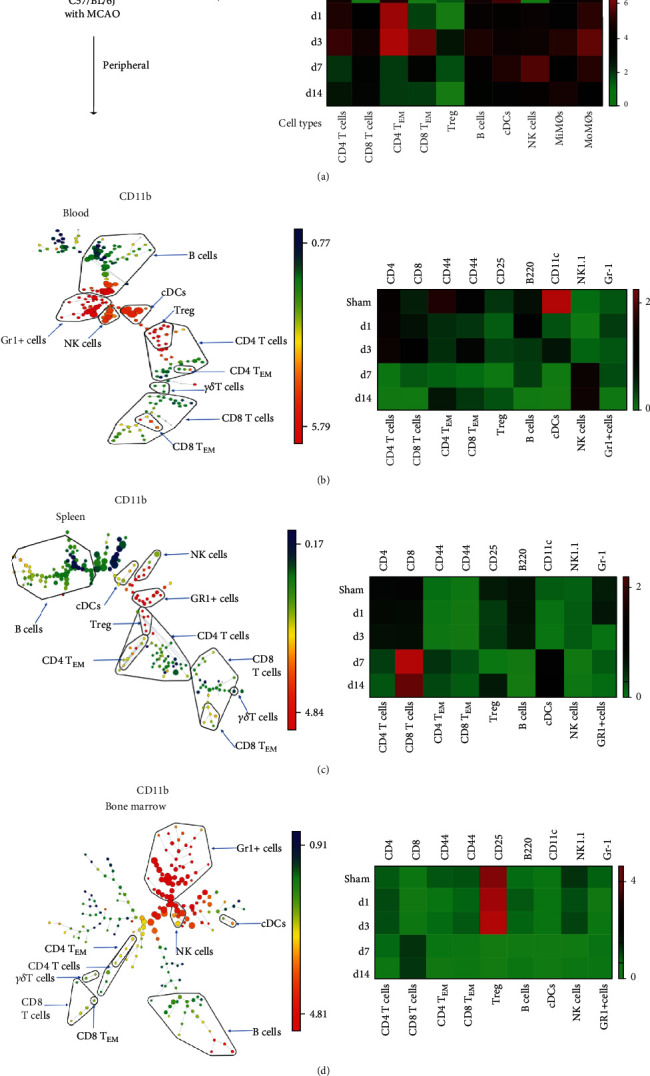
Cell surface markers significantly expressed in different immune cell subtypes from samples of sham, day 1, day 3, day 7, and day 14 poststroke in the brain, blood, spleen, and bone marrow. (a) The surface marker expression levels of specific immune cells according to their cell types in the ischemic brain. The heat map depicts the ArcSinh ratio of median values. The minimum value in each protein expression was used as a control. The cell surface markers include CD45 expression on MiMØs and MoMØs; B220 expression on B cells; CD4 expression on CD4^+^ T cells; CD8 expression on CD8^+^ T cells; CD44 expression on CD4 T_EM_ and CD8 T_EM_ cells; CD25 expression on Tregs; CD11c expression on cDCs; NK1.1 expression on NK cells; and Gr-1 expression on Gr-1^+^ cells. (b–d) Illustrative SPADE tree to present the identified immune cell types in the peripheral blood (b), spleen (c), and bone marrow (d) (left). The cell surface marker expression levels of specific immune cells' on the corresponding immune cell types (right).

**Figure 4:**
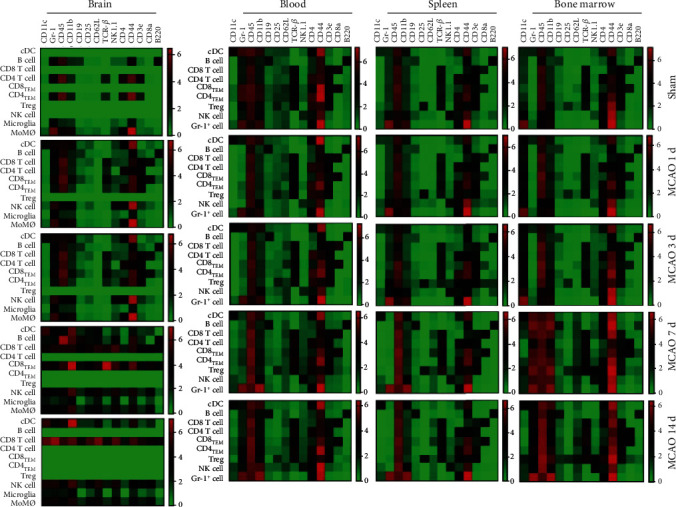
The surface marker expression levels of specific immune cells on corresponding cell types. The expressions of CD11c, Gr-1, CD45, CD11b, CD19, CD25, CD62L, TCR-beta, NK1.1, CD4, CD44, CD3e, CD8a, and B220 in various leukocytes, including cDC, B cell, CD8 T cell, CD4 T cell, CD8 T_EM_, CD4 T_EM_, Treg, and NK cell, in the ischemic brain, peripheral blood, spleen, and bone marrow were measured from samples of sham, day 1, day 3, day 7, and day 14 poststroke in the brain, blood, spleen, and bone marrow. The expression of CD62L has significant increases at day 7 in NK cells, CD8 T cells, microglia, and monocyte-derived macrophages (MiMØs and MoMØs) in the brain tissue.

Our research also revealed that the expression levels of cell surface markers changed in various leukocytes across different time points (Figures 3 and 4). The most dynamic changes of those surface marker expressions happened in the ischemic brain. CD4 and CD8 had higher expressions from day 1 to day 3 (the acute phase) than day 7 and day 14 (subacute phase), while the CD45 expression of both MiMØs and MoMØs kept a relatively higher level over the whole period (Figure 3). Cell surface markers are mainly used to identify leukocyte phenotypes, but their expression levels also reveal the maturation state and functions of individual cells. For instance, Itoh et al. showed that CD4 expression levels correlated with Th cell polarization; specifically, Th2 cells express less CD4 compared to Th1 cells [28]. In our current study, compared to sham control, we found that CD4 expressions in the acute phase (day 1 and day 3) were higher than the expression in the subacute phase (day 7 and day 14), suggesting that in the acute phase, the CD4 T cells are more likely to polarize to Th1 status in the proinflammation stage (Figure 3). Others also reported that stroke resulted in increased CD45 expression intensity shifting from low to medium 24 h after stroke onset [29]. Our results showed that CD45 expressions increased in both MiMØs and MoMØs in the ischemic brain after stroke. Furthermore, it was reported that CD11b expression increased in microglia after stroke [26]. Our data showed that CD11b expression increased after stroke compared to the sham group (Figure 3).

Interestingly, our data showed that the CD62L expression in the brain had significantly increased in NK cells, CD8 T cells, microglia, and monocyte-derived macrophages (MoMØs) at day 7 (Figure 4). CD62L, also known as L-selectin (SELL), is a key adhesion molecule that regulates both the leukocyte migration at the inflammation sites and the lymphocyte recirculation between the blood and lymphoid tissues. It is commonly accepted that stroke-induced brain damage causes neuronal cell death and the release of damage-associated molecular patterns (DAMPs), which initiates the innate and adaptive immune response [30]. However, it is still not clear which factor facilitates the relative long-term immune response [30]. We assumed that the homing immune cell molecule CD62L may have worked as the “tracker” to attract specific immune cells from the peripheral immune cell migration into the brain. Specifically, CD62L acts as a “homing receptor” to enter the secondary lymphoid tissues via high endothelial venues [31]. Interestingly, in our current study, the CD62L significantly increased in day 7 after stroke on set, while the total immune cell numbers in brain peaks on day 3. This phenomenon suggests that in the stroke-induced neuroinflammation, CD62L may track some specific, not all of, immune cell types. Specifically, in 2019, Ito et al. showed that the Tregs had a consistent increase, especially after day 5 [32]. In addition, in our current data, we showed that there was a constant decrease of CD4 Tregs in the peripheral blood, which further supports with Ito et al.'s data that more Treg cells have migrated into the brain. Taken together, our results confirmed that stroke resulted in dynamic changes in many cell surface markers in various leukocytes after stroke. The cell surface marker expression levels could reveal the immune responses after stroke.

### 3.3. Computational Analysis of Immune Cell Networks from Different Tissues at Various Time Points

**Figure 5:**
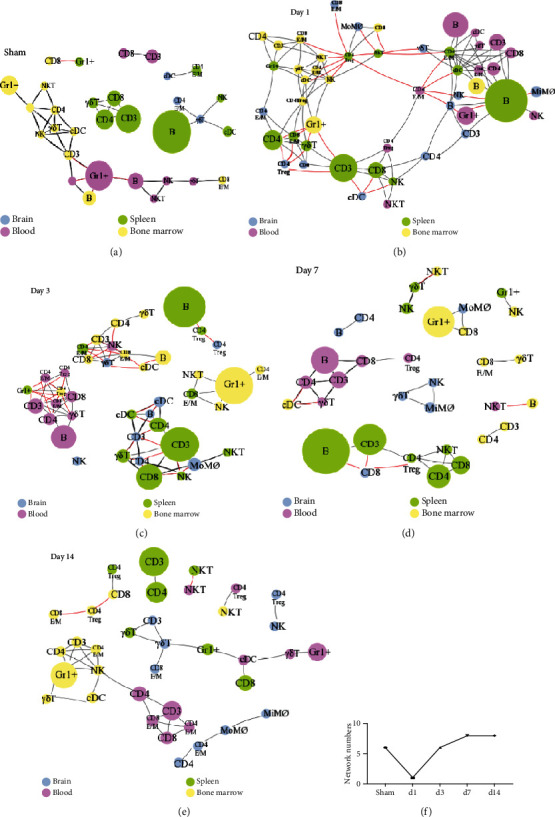
R programming analyses for various immune cell correlations among different organs after stroke. The leukocyte samples were isolated from the brain, blood, spleen, and bone marrow from animals euthanized at day 1, day 3, day 7, and day 14 poststroke. The correlations were analyzed based on cell numbers by using R programming language. Blue circles represent the immune cells from the brain; purple, green, and yellow represent the immune cells from the blood, spleen, and bone marrow, respectively. The red line represents a positive correlation while the black line represents a negative correlation. Immune cell networks present at different time points: (a) sham; (b) day 1; (c) day 3; (d) day 7; (e) day 14. (f) Network numbers from different days after stroke and sham animals are depicted.

Based on the cell numbers, we described the correlation between different cell types among various tissues at different time points using the R program. The red lines indicate the positive correlations between different immune cell types, while the black lines show the negative correlations. This computational analysis predicts the possible immune cell relationships and the networks among the brain, peripheral blood, spleen, and bone marrow (Figure 5). In sham animals, the analysis shows that there are interactions between immune cells from the bone marrow (yellow circles) and blood (purple circles), but most immune cells mainly grouped within each tissue (Figure 5(a)). However, the dramatic changes happened at day 1 after stroke, when the peripheral immune cells migrated into the brain. All cell types from the different organs are grouped as one big network (Figure 5(b)), indicating that all cell types interact with each other among the ischemic brain, peripheral blood, spleen, and bone marrow. Nevertheless, the single, big network observed at day 1 is broken down into 6 small networks at day 3. As a result, immune cells mainly formed networks within their original organs at day 3, and the correlations across different organs are dramatically reduced. However, a significant network between the brain and the spleen was detected, likely indicating that the immune cells from the spleen still affect or interact with the immune components in the brain in a longer time frame (Figure 5(c)).

To explore the interactions among various immune cells across different time courses, we analyzed the network via different time points. We showed a significant brain-spleen interaction, especially in the acute phase (Figure 5). This result is consistent with previous studies that spleen-derived proinflammatory cells move into the peripheral circulation and subsequently migrate into the ischemic brain [33]. The major immune cell types involved in the brain-blood interaction are Gr1^+^ cells and CD3 T cells, including both CD4 and CD8 T cells (Figure 5(c)). In addition, some limited interactions between the bone marrow, blood, and spleen were observed (Figure 5(c)). This interaction reduction trend continues to decrease from day 7 to day 14 after stroke (Figures 5(d) and 5(e)). The numbers of individual networks from sham animals and animals surviving at different days after stroke were portrayed (Figure 5(f)), indicating that the correlations between immune cell types across different organs increased at day 1 and decreased from day 3 to day 14 after stroke.

In conclusion, our study provides a systematic characterization of the immune cells among different tissues and organs from the acute to chronic phase after ischemic stroke in mice. However, there are also some limitations in our current study. For instance, adding functional cell markers into the antibody panel could show the functional changes of various immune cells. Nevertheless, this current study shows a systematic overview of cell populations and provides more information in terms of cell number, cell-cell network, and phenotypes.

## Figures and Tables

**Table 1:** Antibodies used in this study.

Antibodies	Clone	Metal label
Anti-mouse Gr-1	IA8	141Pr
Anti-mouse CD11c	N418	142Nd
Anti-mouse CD69	H1.2F3	145Nd
Anti-mouse CD45	30-F11	147Sm
Anti-mouse CD11b	M1/70	148Nd
Anti-mouse B220	RA3-6B2	176Yb
Anti-mouse CD25	PC61	151Eu
Anti-mouse CD3	145.2C11	152Sm
Anti-mouse Ter119	Ter119	154Sm
Anti-mouse CD62L	MEL-14	160Gd
Anti-mouse CD8	53-6.7	168Er
Anti-mouse TCR*β*	H57-597	169Tm
Anti-mouse NK1.1	PK136	170Er
Anti-mouse CD44	IM7	171Yb
Anti-mouse CD4	RM4-5	172Yb
DNA staining		191/193Ir
Live/dead sating		195Pt
Barcoding		102-110Pd

**Table 2:** Identified immune cell types and their identifying markers.

Cell types	Defined markers
Monocyte derived macrophages (MoMØs)	CD45^hi^CD11b^+^
Microglia derived macrophages (MiMØs)	CD45^lo^CD11b^+^
*αβ*T cells	TCR*β*^+^CD3^+^
*γδ*T cells	TCR*β*^−^CD3^+^
B cells	CD45^+^B220^+^
CD8 T cells	CD45^+^ CD8^+^
CD4 T cells	CD45^+^ CD4^+^
Treg cells	CD3^+^CD4^+^ CD25^+^
CD4 T_EM_	CD3^+^CD4^+^CD44^+^
CD8 T_EM_	CD3^+^CD8^+^CD44^+^
NK cells	CD45^+^ NK1.1^+^

## Data Availability

The additional data for CyTOF are provided in supplementary materials.

## Supplementary Materials

Supplementary Materials Supplementary Figure 1: representative SPADE and viSNE figures for cell-type identification in peripheral blood and major cell surface markers used for cell identification including CD45, CD11b, CD11c, Gr1, B220, NK1.1, CD3, CD8, CD4, CD44, CD25, and TCR*β*. Illustrative SPADE tree (top) viSNE (bottom) to present identified immune cell types in peripheral blood. In SPADE, the colored standards present the expression levels of markers' median, and the node sizes show the numbers of cells. In viSNE, the color gradients show the markers' intensity, and each dot presents a single cell. Supplementary Figure 2: representative SPADE and viSNE figures for cell type identification in blood and major cell surface markers used for cell identification including CD45, CD11b, CD11c, Gr1, B220, NK1.1, CD3, CD8, CD4, CD44, CD25, and TCR*ß*. Illustrative SPADE tree (top) viSNE (bottom) to present identified immune cell type in the spleen. In SPADE, the colored standards present the expression levels of markers' median, and the node sizes show the numbers of cells. In viSNE, the color gradients show the markers' intensity, and each dot presents a single cell. Supplementary Figure 3: representative SPADE and viSNE figures for cell-type identification in the bone marrow and major cell surface markers used for cell identification including CD45, CD11b, CD11c, Gr1, B220, NK1.1, CD3, CD8, CD4, CD44, CD25, and TCR*ß*. Illustrative SPADE tree (top) viSNE (bottom) to present identified immune cell type in the bone marrow. In SPADE, the colored standards present the expression level of markers' median, and the node sizes show the numbers of cells. In viSNE, the color gradients show the markers' intensity, and each dot presents a single cell.

## References

[1] Wang C. W., Su L. L., Hua Q. J. (2018). Aspirin resistance predicts unfavorable functional outcome in acute ischemic stroke patients. *Brain Research Bulletin*.

[2] Gu L. J., Xiong X. X., Ito T. (2014). Moderate hypothermia inhibits brain inflammation and attenuates stroke-induced immunodepression in rats. *CNS Neuroscience & Therapeutics*.

[3] Xiong X., Gu L., Wang Y. (2016). Glycyrrhizin protects against focal cerebral ischemia via inhibition of T cell activity and HMGB1-mediated mechanisms. *Journal of Neuroinflammation*.

[4] Chamorro Á., Meisel A., Planas A. M., Urra X., van de Beek D., Veltkamp R. (2012). The immunology of acute stroke. *Nature Reviews. Neurology*.

[5] Iadecola C., Anrather J. (2011). The immunology of stroke: from mechanisms to translation. *Nature Medicine*.

[6] Korhonen P., Kanninen K. M., Lehtonen Š. (2015). Immunomodulation by interleukin-33 is protective in stroke through modulation of inflammation. *Brain, Behavior, and Immunity*.

[7] Xiong X., Gu L., Zhang H., Xu B., Zhu S., Zhao H. (2013). The protective effects of T cell deficiency against brain injury are ischemic model-dependent in rats. *Neurochemistry International*.

[8] Fan Y., Xiong X., Zhang Y. (2016). MKEY, a peptide inhibitor of CXCL4-CCL5 heterodimer formation, protects against stroke in mice. *Journal of the American Heart Association*.

[9] Seifert H. A., Hall A. A., Chapman C. B., Collier L. A., Willing A. E., Pennypacker K. R. (2012). A transient decrease in spleen size following stroke corresponds to splenocyte release into systemic circulation. *Journal of Neuroimmune Pharmacology*.

[10] Adeoye O., Walsh K., Woo J. G. (2014). Peripheral monocyte count is associated with case fatality after intracerebral hemorrhage. *Journal of Stroke and Cerebrovascular Diseases*.

[11] Cortina M. G., Campello A. R., Conde J. J. (2008). Monocyte count is an underlying marker of lacunar subtype of hypertensive small vessel disease. *European Journal of Neurology*.

[12] Walsh K. B., Sekar P., Langefeld C. D. (2015). Monocyte count and 30-day case fatality in intracerebral hemorrhage. *Stroke*.

[13] Wang Y., Luo Y., Yao Y. (2020). Silencing the lncRNA *Maclpil* in pro-inflammatory macrophages attenuates acute experimental ischemic stroke via LCP1 in mice. *Journal of Cerebral Blood Flow & Metabolism*.

[14] Benakis C., Brea D., Caballero S. (2016). Commensal microbiota affects ischemic stroke outcome by regulating intestinal *γδ* T cells. *Nature Medicine*.

[15] Wang Y., Jin H., Wang W., Wang F., Zhao H. (2019). Myosin1f-mediated neutrophil migration contributes to acute neuroinflammation and brain injury after stroke in mice. *Journal of Neuroinflammation*.

[16] Korin B., Dubovik T., Rolls A. (2018). Mass cytometry analysis of immune cells in the brain. *Nature Protocols*.

[17] Joo S. P., Xie W., Xiong X., Xu B., Zhao H. (2013). Ischemic postconditioning protects against focal cerebral ischemia by inhibiting brain inflammation while attenuating peripheral lymphopenia in mice. *Neuroscience*.

[18] Zunder E. R., Finck R., Behbehani G. K. (2015). Palladium-based mass tag cell barcoding with a doublet-filtering scheme and single-cell deconvolution algorithm. *Nature Protocols*.

[19] Kotecha N., Krutzik P. O., Irish J. M. (2010). Web-based analysis and publication of flow cytometry experiments. *Current Protocols in Cytometry*.

[20] Qiu P., Simonds E. F., Bendall S. C. (2011). Extracting a cellular hierarchy from high-dimensional cytometry data with SPADE. *Nature Biotechnology*.

[21] Korin B., Ben-Shaanan T. L., Schiller M. (2017). High-dimensional, single-cell characterization of the brain's immune compartment. *Nature Neuroscience*.

[22] Amir E.-a. D., Davis K. L., Tadmor M. D. (2013). viSNE enables visualization of high dimensional single-cell data and reveals phenotypic heterogeneity of leukemia. *Nature Biotechnology*.

[23] Elkind M. S. V., Sciacca R. R., Boden-Albala B., Rundek T., Paik M. C., Sacco R. L. (2005). Relative elevation in baseline leukocyte count predicts first cerebral infarction. *Neurology*.

[24] Grau A. J., Boddy A. W., Dukovic D. A. (2004). Leukocyte count as an independent predictor of recurrent ischemic events. *Stroke*.

[25] Ren H., Liu X., Wang L., Gao Y. (2017). Lymphocyte-to-monocyte ratio: a novel predictor of the prognosis of acute ischemic stroke. *Journal of Stroke and Cerebrovascular Diseases*.

[26] Liu Z. J., Chen C., Li F. W. (2015). Splenic responses in ischemic stroke: new insights into stroke pathology. *CNS Neuroscience & Therapeutics*.

[27] Ajmo C. T., Vernon D. O. L., Collier L. (2008). The spleen contributes to stroke-induced neurodegeneration. *Journal of Neuroscience Research*.

[28] Itoh Y., Wang Z., Ishida H. (2005). Decreased CD4 expression by polarized T helper 2 cells contributes to suboptimal TCR‐induced phosphorylation and reduced Ca^2+^ signaling. *European Journal of Immunology*.

[29] Denker S. P., Ji S., Dingman A. (2007). Macrophages are comprised of resident brain microglia not infiltrating peripheral monocytes acutely after neonatal stroke. *Journal of Neurochemistry*.

[30] Shi K., Tian D. C., Li Z. G., Ducruet A. F., Lawton M. T., Shi F. D. (2019). Global brain inflammation in stroke. *The Lancet Neurology*.

[31] Perfilyeva Y. V., Ostapchuk Y. O., Abdolla N., Tleulieva R., Krasnoshtanov V. C., Belyaev N. N. (2019). Exogenous melatonin up-regulates expression of CD62L by lymphocytes in aged mice under inflammatory and non-inflammatory conditions. *Immunological Investigations*.

[32] Ito M., Komai K., Mise-Omata S. (2019). Brain regulatory T cells suppress astrogliosis and potentiate neurological recovery. *Nature*.

[33] Pennypacker K. R., Offner H. (2014). The role of the spleen in ischemic stroke. *Journal of Cerebral Blood Flow and Metabolism*.

