# Supplementary material for: Systematic Study of the Immune Components after Ischemic Stroke Using CyTOF Techniques
Source: J Immunol Res. 2020 Aug 28;2020:9132410. doi: 10.1155/2020/9132410 (PMC7474762; doi:10.1155/2020/9132410)
Supplement: Supplementary Materials — Supplementary Figure 1: representative SPADE and viSNE figures for cell-type identification in peripheral blood and major cell surface markers used for cell identification including CD45, CD11b, CD11c, Gr1, B220, NK1.1, CD3, CD8, CD4, CD44, CD25, and TCRβ. Illustrative SPADE tree (top) viSNE (bottom) to present identified immune cell types in peripheral blood. In SPADE, the colored standards present the expression levels of markers' median, and the node sizes show the numbers of cells. In viSNE, the color gradients show the markers' intensity, and each dot presents a single cell. Supplementary Figure 2: representative SPADE and viSNE figures for cell type identification in blood and major cell surface markers used for cell identification including CD45, CD11b, CD11c, Gr1, B220, NK1.1, CD3, CD8, CD4, CD44, CD25, and TCRß. Illustrative SPADE tree (top) viSNE (bottom) to present identified immune cell type in the spleen. In SPADE, the colored standards present the expression levels of markers' median, and the node sizes show the numbers of cells. In viSNE, the color gradients show the markers' intensity, and each dot presents a single cell. Supplementary Figure 3: representative SPADE and viSNE figures for cell-type identification in the bone marrow and major cell surface markers used for cell identification including CD45, CD11b, CD11c, Gr1, B220, NK1.1, CD3, CD8, CD4, CD44, CD25, and TCRß. Illustrative SPADE tree (top) viSNE (bottom) to present identified immune cell type in the bone marrow. In SPADE, the colored standards present the expression level of markers' median, and the node sizes show the numbers of cells. In viSNE, the color gradients show the markers' intensity, and each dot presents a single cell. [file 9132410.f1.pdf]

Supplementary Materials

The sample from peripheral blood, spleen and bone marrow were also analyzed using both viSNE and SPADE separately. The viSNE presents a two-dimensional data; each individual cell shows a scatter plot to present the labeled marker expression level for each cell. SPADE organizes cells into a hierarchy based on the related phenotype markers to identify cell types and shows the cellular heterogeneity Supplementary Figures (1-3).

Characterization of the immune cells from blood (supplementary figure 1), spleen (supplementary figure 2) and bone marrow (supplementary figure 3) by mass cytometry using SPADE (top), and viSNE (bottom).

Supplementary Figure 1 Blood

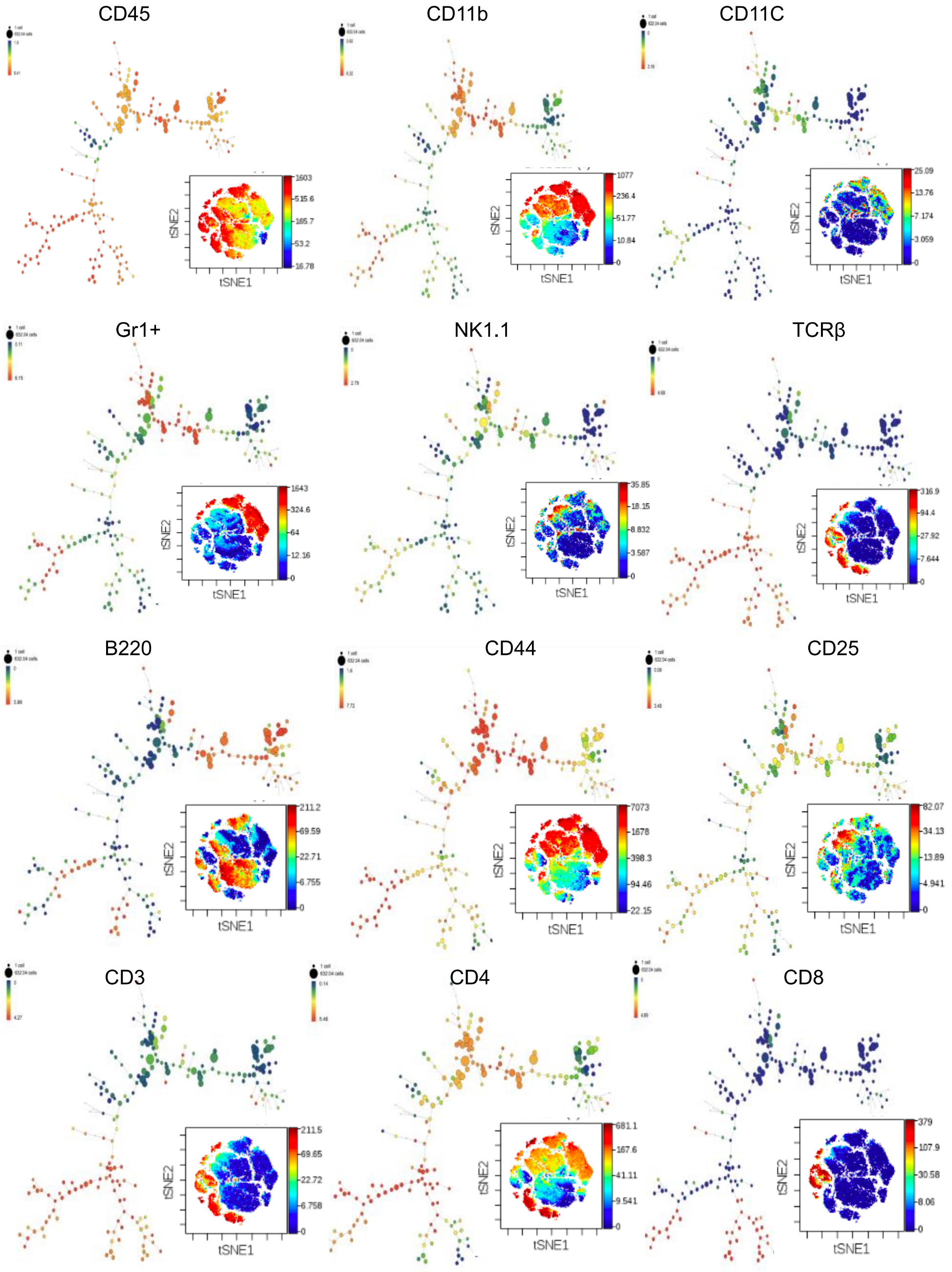

Supplementary Figure 1: Representative SPADE and ViSNE figures for cell type identification in peripheral blood, major cell surface markers used for cell identification including CD45, CD11b, CD11c, Gr1, B220, NK1.1, CD3, CD8, CD4, CD44, CD25 and TCRß. Illustrative SPADE tree (Top) ViSNE (bottom) to present identified immune cell types in peripheral blood. In SPADE, the colored standards present the expression levels of markers' median, and the node sizes shows the numbers of cells. In ViSNE, the color gradients show the markers' intensity, and each dot presents a single cell.

Supplementary Figure2 Spleen

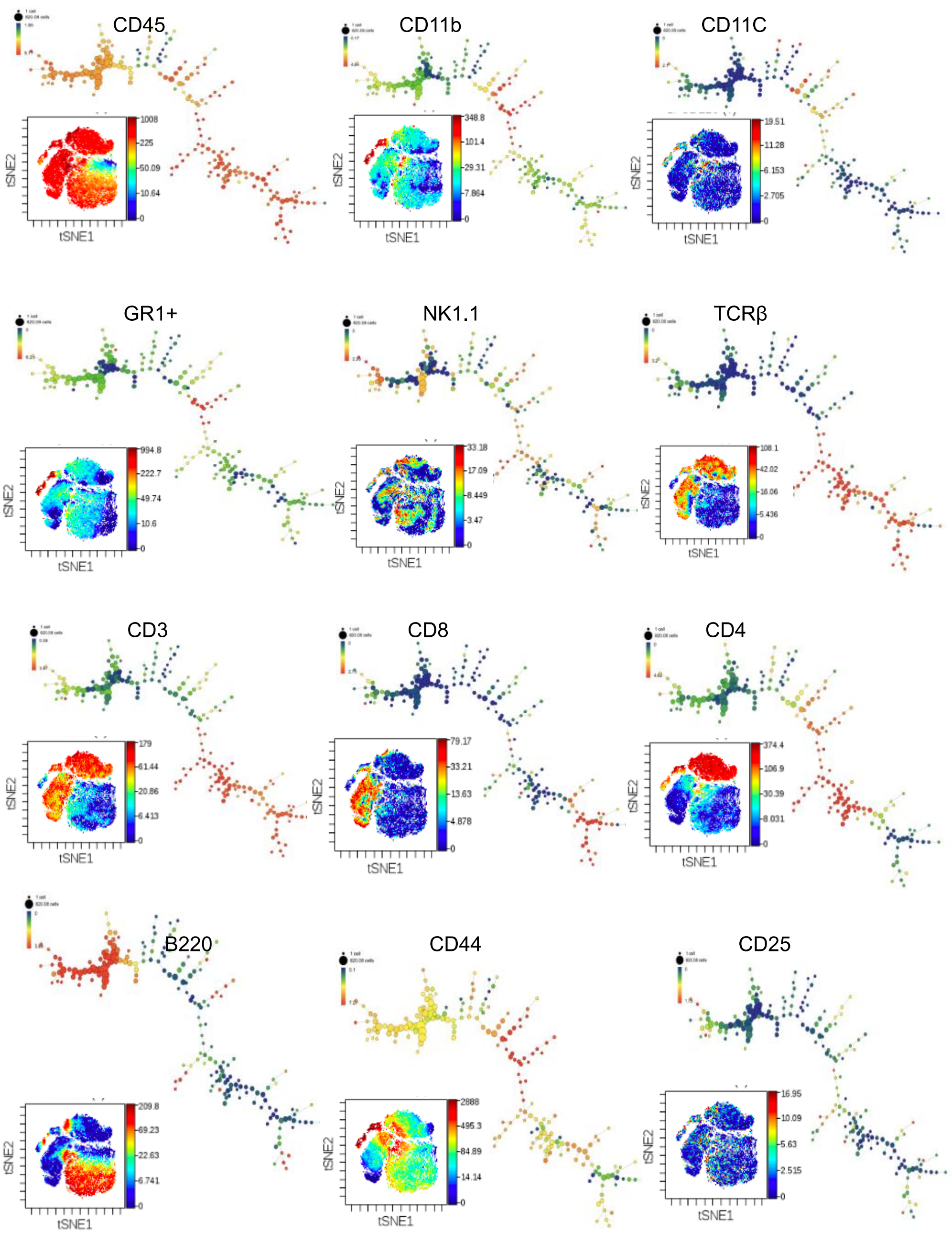

Supplementary Figure 2: Representative SPADE and ViSNE figures for cell type identification in blood, major cell surface markers used for cell identification including CD45, CD11b, CD11c, Gr1, B220, NK1.1, CD3, CD8, CD4, CD44, CD25 and TCRß Illustrative SPADE tree (Top) ViSNE (bottom) to present identified immune cell type in the spleen. In SPADE, the colored standards present the expression levels of markers' median, and the node sizes shows the numbers of cells. In ViSNE, the color gradients show the markers' intensity, and each dot presents a single cell.

Supplementary Figure3 Bone marrow

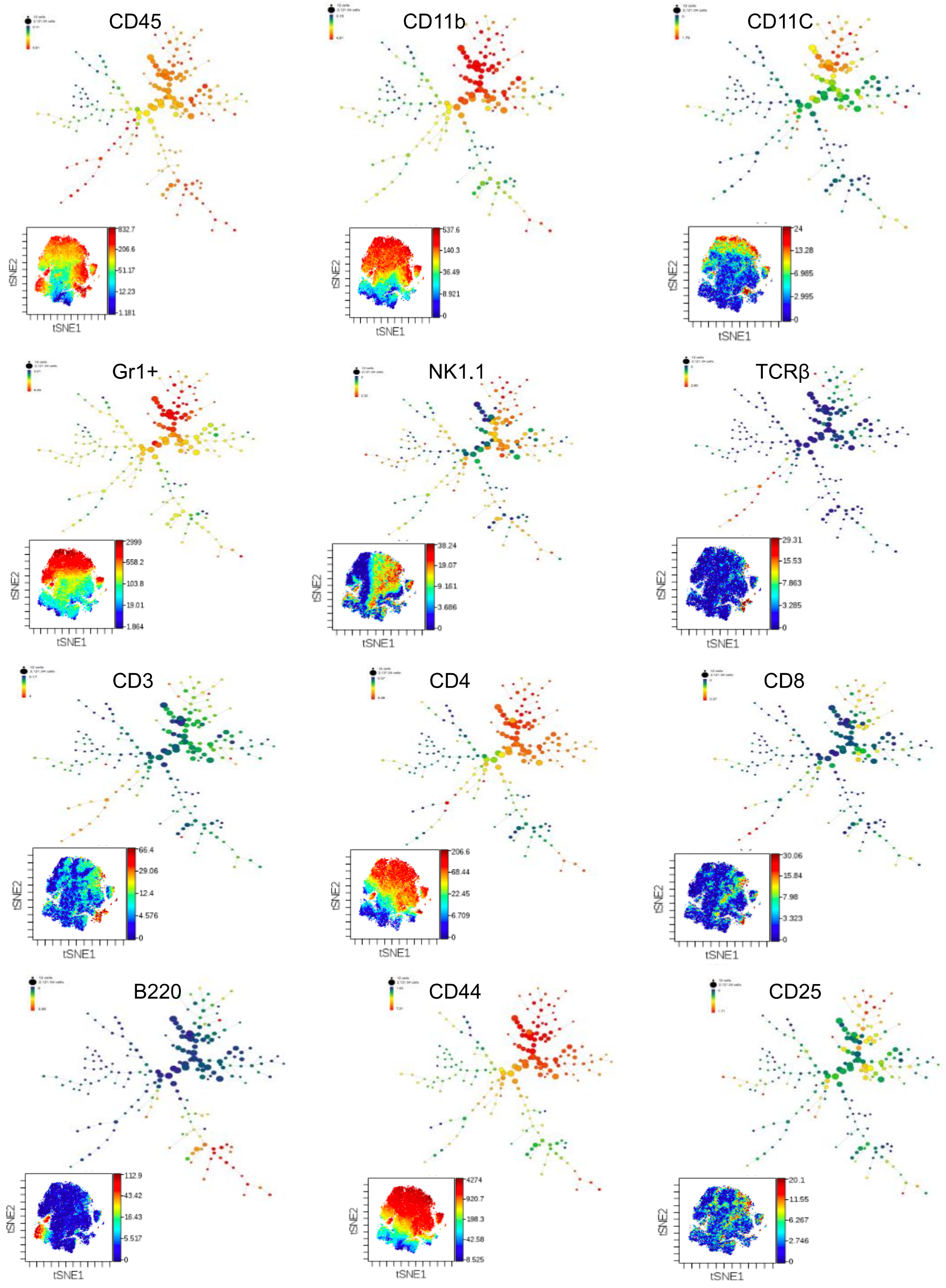

Supplementary Figure 3: Representative SPADE and ViSNE figures for cell type identification in bone marrow, major cell surface markers used for cell identification including CD45, CD11b, CD11c, Gr1, B220, NK1.1, CD3, CD8, CD4, CD44, CD25 and TCR ß Illustrative SPADE tree (Top) ViSNE (bottom) to present identified immune cell type in bone marrow. In SPADE, the colored standards present the

expression level of markers' median, and the node sizes shows the numbers of cells. In ViSNE, the color gradients show the markers' intensity, and each dot presents a single cell.
